# An Unusual Presentation of Fentanyl-Induced Rhabdomyolysis

**DOI:** 10.7759/cureus.83426

**Published:** 2025-05-03

**Authors:** Olivia D Keenum, Shivam Patel, Arthi Balasubramaniam, William Bates, Donald Smallwood

**Affiliations:** 1 Radiology, Medical College of Georgia at Augusta University, Augusta, USA

**Keywords:** compartment syndrome, computed tomography angiography (cta), computed tomography (ct), fentanyl, magnetic resonance imaging (mri), myositis, rhabdomyolysis

## Abstract

Rhabdomyolysis is a pathological condition characterized by the breakdown of muscle tissue, which often presents with nonspecific imaging findings but necessitates prompt intervention. Providing a differential diagnosis based on imaging findings can significantly aid managing physicians, while a clear clinical context enhances the radiologist's ability to identify critical findings. In this report, we present a case of proximal lower extremity fentanyl-induced rhabdomyolysis identified on computed tomography (CT) angiography (CTA) performed to evaluate for compartment syndrome in the distal left lower extremity. We will discuss the expected imaging findings associated with rhabdomyolysis on both CT and magnetic resonance imaging (MRI).

## Introduction

Radiologists play a crucial role in identifying imaging findings to guide patient diagnosis and care. This case describes rhabdomyolysis seen on a computed tomography (CT) angiography (CTA) conducted to evaluate for compartment syndrome of the left lower extremity. Rhabdomyolysis is a condition resulting from the destruction of a large number of myocytes, often from extreme exercise, injury, long periods of immobility, and even extensive burns. The mortality rate in rhabdomyolysis is around 10% but jumps as high as 50% with acute kidney injury (AKI) and creatine kinase (CK) above 50,000 U/L [1]. Imaging findings associated with rhabdomyolysis are nonspecific and may overlap with other conditions that cause acute myositis, so familiarity with the clinical context is important for accurate imaging interpretation. While magnetic resonance imaging (MRI) demonstrates greater sensitivity for detecting myositis, it lacks increased specificity. Clinical context and laboratory studies are the foundation of the diagnosis and treatment of rhabdomyolysis, but imaging occasionally informs diagnosis and decision-making.

## Case presentation

A 22-year-old female presented to the emergency department after reportedly snorting fentanyl, mistakenly believing it to be Roxicodone. Emergency medical services found her distressed with “altered mental status.” She was hypotensive (87/58 mmHg) and was stabilized with intravenous lactated Ringer's solution and pressor agents. She reported diminished sensation and paresthesia in her lower extremities, more pronounced on the left side.

By the time she was admitted to the hospital, her condition had worsened to include diffuse body pain and loss of sensation and motor function in her left ankle and toes. She retained motor function in her left hip and entire right lower extremity. The patient denied intravenous drug use, incontinence, previous neuropathy, and complete review of systems (ROS) was otherwise negative. Her only prescribed medication was intramuscular medroxyprogesterone, administered every three months.

Initial laboratory evaluation revealed leukocytosis, significantly elevated CK, and electrolyte disturbances, including hyponatremia, hyperkalemia, hypochloremia, and hyperphosphatemia (Table 1). Metabolic acidosis was suggested by a bicarbonate of 11 mEq/L. Transaminases were elevated along with alkaline phosphatase (Table 1). Acute kidney injury (AKI) was also indicated by elevated creatinine (Table 1). Urinalysis was positive for both blood and red blood cells. The urine drug screen was positive only for fentanyl. The patient was monitored with telemetry while her electrolyte disturbances were corrected with continued intravenous fluids, calcium gluconate, insulin with dextrose, and sodium bicarbonate.

**Table 1:** Referenced serum laboratory values The patient’s results are noted in the middle column with the reference ranges in the right-hand column. Serum reference values are from the American Board of Internal Medicine [2].

Serum laboratory test	Patient’s value	Reference range
White blood cell count (WBC)	31,000 cells/μL	4,000-11,000/μL
Creatine kinase (CK)	125,334 U/L	Female: 30-135 U/L; Male: 55-170 U/L
Sodium (Na)	125 mEq/L	136-145 mEq/L
Potassium (K)	6.7 mEq/L	3.5-5.0 mEq/L
Chloride (Cl)	86 mEq/L	98-106 mEq/L
Phosphorus (Phos)	5.2 mg/dL	3.0-4.5 mg/dL
Bicarbonate (CO_2_)	11 mEq/L	23-28 mEq/L
Aspartate aminotransferase (AST)	339 U/L	10-40 U/L
Alanine aminotransferase (ALT)	200 U/L	10-40 U/L
Alkaline phosphatase (ALK)	132 U/L	30-120 U/L
Creatinine (Cr)	2.42 mg/dL	Female: 0.50-1.10 mg/dL; Male: 0.70-1.30 mg/dL

**Figure 1:**
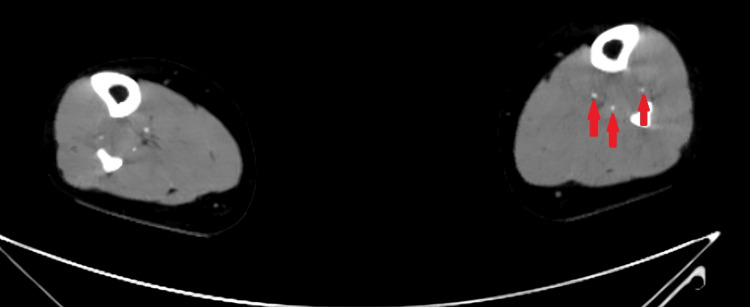
CT angiogram of distal bilateral lower extremities Arterial phase of axial post-contrast CT demonstrates that all three major arteries of the bilateral lower extremities just proximal to the ankles are patent. Arrows mark the three arteries in the left lower extremity, which is the symptomatic limb.

**Figure 2:**
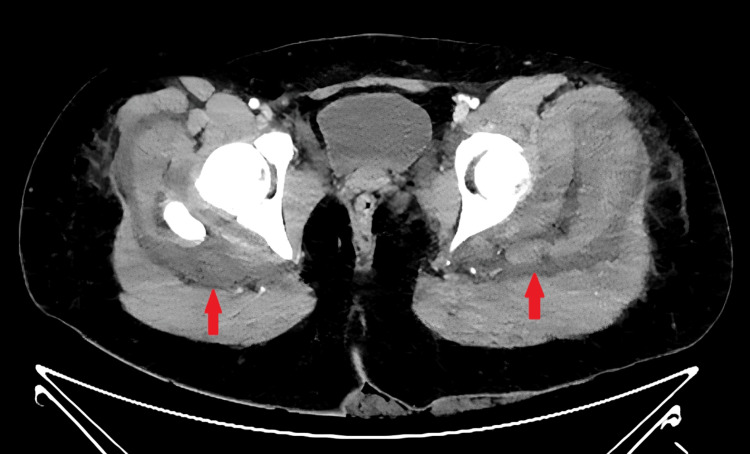
Axial post-contrast CT of the proximal lower extremity musculature Axial contrast-enhanced CT demonstrates bilateral gluteal muscle enlargement, muscle edema, and heterogeneous muscle enhancement. Arrows mark hypodense areas within the heterogeneous enhancement of the muscles.

**Figure 3:**
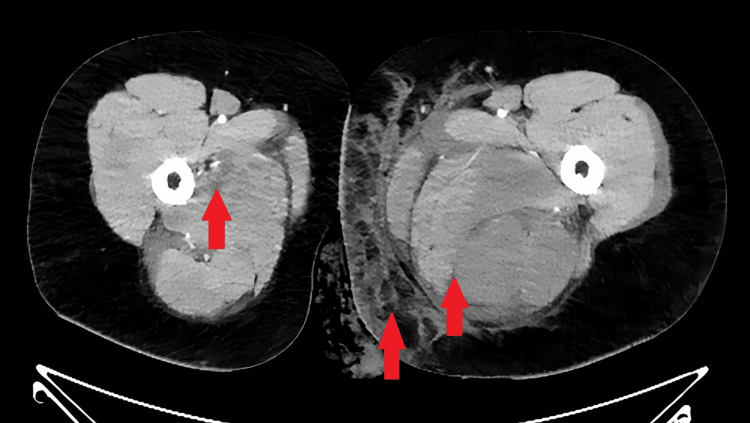
Axial post-contrast CT of the bilateral thighs Axial contrast-enhanced CT shows left greater than right thigh muscle enlargement, muscle edema, and heterogeneous muscle enhancement, most pronounced in the bilateral adductor and posterior thigh compartment musculature. Marked inflammatory changes and edema are also demonstrated within the left medial thigh subcutaneous soft tissues. Arrows mark some of the heterogeneous enhancement and muscle edema in each extremity, as well as inflammatory changes in the subcutaneous tissue of the left thigh.

**Figure 4:**
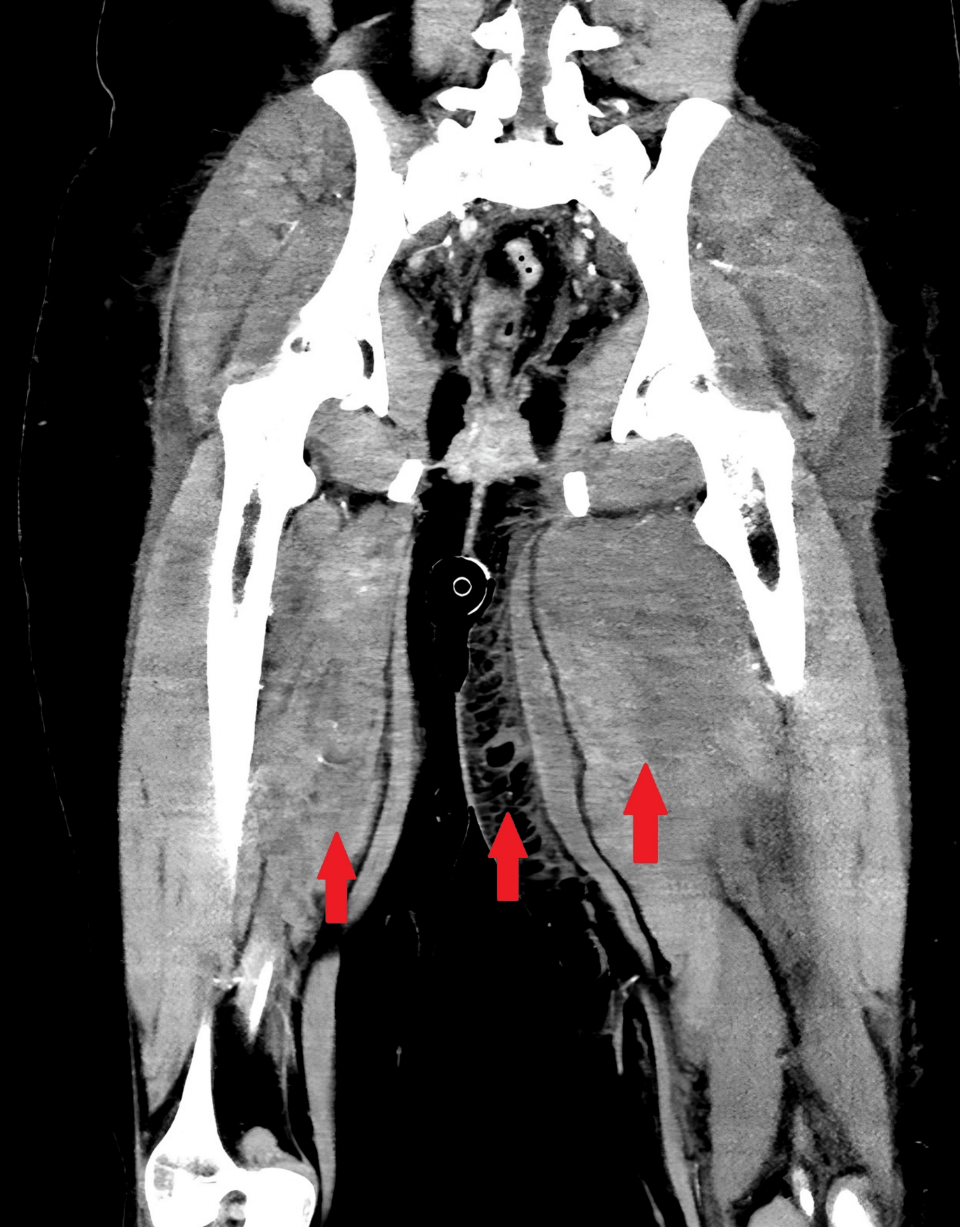
Coronal post-contrast CT of the proximal bilateral lower extremities Coronal contrast-enhanced CT shows bilateral, left greater than right, hip, and thigh muscle heterogeneous enhancement with muscle enlargement. Arrows mark areas of heterogenous enhancement and muscle enlargement bilaterally as well as inflammatory changes of the subcutaneous tissue of the thigh noted in the preceding figure.

On day 2, vascular surgery was consulted due to concern for possible compartment syndrome in the left lower extremity. After examining the patient, they recommended a CTA for further evaluation, which revealed that all major arteries of the bilateral lower extremities were patent (Figure 1). However, hypoattenuation was observed in the proximal lower extremity muscles bilaterally, accompanied by heterogeneous enhancement on the post-contrast images. Furthermore, significant inflammatory changes were noted in the left medial thigh, extending to the knee (Figures 2-4). These findings were present proximal to the patient’s symptoms in the distal extremity.

The patient was discharged on day 4 after receiving 10 total liters of lactated Ringer's solution. At discharge, she was hemodynamically stable, renal function had normalized, leukocytosis had resolved, and both transaminase and CK levels continued to trend downward. However, the patient reported ongoing weakness in the left ankle. A neurology follow-up was scheduled, but no records indicate that she made it to the appointment.

## Discussion

Rhabdomyolysis is characterized by the release of muscle myocyte contents into the bloodstream following muscle injury, potentially causing electrolyte imbalances and AKI [3]. Our patient presented with hypotension and left lower extremity weakness, pain, and paresthesia following fentanyl ingestion. Laboratory findings, including markedly elevated CK, hyperkalemia, hyperphosphatemia, and metabolic acidosis, supported the diagnosis of rhabdomyolysis (Table 1) [3,4]. The consensus for CK levels to be diagnostic of rhabdomyolysis is typically 10 times the upper limit of normal, and our patient reached over 90 times that limit [1]. She also had elevated transaminases, which are common in rhabdomyolysis even without liver injury [5]. Rhabdomyolysis is primarily diagnosed through laboratory testing (elevated CK, myoglobinuria) with a supporting history (mechanism of injury, weakness, myalgias) and clinical presentation (dark urine), although the classic triad of myalgias, weakness, and dark urine is seen in less than 10% of patients [1].

Compartment syndrome can both lead to rhabdomyolysis and emerge as a complication of rhabdomyolysis [6-9]. Compartment syndrome was suspected in our patient due to her weakness, pain, and paresthesia in the setting of rhabdomyolysis, prompting CTA acquisition. CTA helped exclude compartment syndrome and further supported the diagnosis of rhabdomyolysis with findings of muscle injury and inflammation. On CT, rhabdomyolysis may present with marked muscle swelling with possible fascial thickening and fluid along fascial planes. A contrasted CT may demonstrate heterogeneous enhancement in the setting of muscle injury and edema (Figures 2-4), or if progressed to myonecrosis, areas of nonenhancement surrounded by peripheral enhancement [10,11]. In cases of severe muscle injury, hemorrhage may be visualized as areas of hyperdensity. Because there are many etiologies of muscle destruction, which may or may not result in rhabdomyolysis, CT findings are nonspecific. Therefore, CT imaging is recommended only when the etiology of rhabdomyolysis is unclear, a distinct treatable cause is suspected (e.g., fracture), or complications like compartment syndrome need to be ruled out, as in our patient.

MR may be preferable to CT to examine the extent of muscle damage when considering fasciotomy for compartment syndrome [12]. MRI is more sensitive for detecting myositis, but its findings are also nonspecific [13]. Acute myositis typically presents as an iso- to hyperintense signal on T1-weighted MR images and as a hyperintense signal on T2-weighted MR images, including short tau inversion recovery (STIR) [10,13,14]. T1 enhancement can also be seen following gadolinium contrast administration [10,13,14]. Rhabdomyolysis imaging findings can be further broken down into type 1, in which the signal intensity described above is homogenous in the affected muscle, and type 2, in which the signal intensities are heterogenous with rim-enhancing areas on the post-contrast image [10]. Type 1 is thought to be indicative of overexertion, while type 2 is thought to be evidence of progression to myonecrosis, supported by stippling rim enhancement seen on post-contrast imaging with type 2 rhabdomyolysis [10]. With chronic myositis, fatty atrophy of the musculature on T1-weighted MR may be observed, contrasting the acute findings described herein [12].

Notably, our patient’s weakness and paresthesia were distal to the observed radiographic findings. One plausible explanation is that the patient remained immobile following fentanyl ingestion, resulting in ischemic damage to proximal muscle tissue and contributing to rhabdomyolysis. Swelling in the proximal lower extremity musculature may have compressed the sciatic nerve, causing the observed distal weakness and sensory changes [15]. Alternatively, direct pressure on the sciatic nerve may have caused the observed symptoms [16]. Peripheral nerve compression is expected to resolve within weeks to months [17]. If the patient remains weak, physical therapy might be warranted alongside an investigation into other causes of weakness and sensory changes. The patient’s lack of follow-up leaves her long-term recovery and prognosis unclear, underscoring the complexity of diagnosing and managing muscle injuries leading to rhabdomyolysis.

Early recognition of rhabdomyolysis is crucial to prevent complications such as cardiac arrhythmia, seizures, and AKI with prompt fluid resuscitation and careful electrolyte management. Imaging plays a supportive role in diagnosis, but findings are nonspecific, and clinical judgment is critical. In this case, CTA revealed inflammatory muscle changes and muscle injury, which were consistent with rhabdomyolysis when considering the patient’s symptoms and laboratory evaluation.

## Conclusions

Rhabdomyolysis occurs when injured myocytes spill their contents into the bloodstream, which can lead to AKI and life-threatening electrolyte imbalances. Early diagnosis and intervention are essential to mitigate complications like arrhythmia, seizure, and kidney damage, and imaging should be used judiciously to guide treatment decisions. Imaging can help confirm muscle injury and can assist in excluding suspected complications, such as compartment syndrome, but is nonspecific for rhabdomyolysis. In this case, CTA identified muscle injury but did not correlate in location with the patient’s symptoms, illustrating the importance of considering the full clinical picture and the full radiologic study.
